# Association of the beta-1 adrenergic receptor carboxyl terminal variants with left ventricular hypertrophy among diabetic and non-diabetic survivors of acute myocardial infarction

**DOI:** 10.1186/1475-2840-9-42

**Published:** 2010-08-23

**Authors:** Anna E Hakalahti, Jari M Tapanainen, Juhani M Junttila, Kari S Kaikkonen, Heikki V Huikuri, Ulla E Petäjä-Repo

**Affiliations:** 1 Institute of Biomedicine, Department of Anatomy and Cell Biology, University of Oulu, P.O. Box 5000, FI-90014 Oulu, Finland; 2 Department of Cardiology, Helsinki University Central Hospital, FI-00290 Helsinki, Finland; 3 Institute of Clinical Medicine, Department of Internal Medicine, Division of Cardiology, University of Oulu, P.O. Box 5000, FI-90014 Oulu, Finland

## Abstract

**Background:**

The beta-1 adrenergic receptor (β_1_AR) plays a fundamental role in the regulation of cardiovascular functions. It carries a nonsynonymous single nucleotide polymorphism in its carboxyl terminal tail (Arg389Gly), which has been shown to associate with various echocardiographic parameters linked to left ventricular hypertrophy (LVH). Diabetes mellitus (DM), on the other hand, represents a risk factor for LVH. We investigated the possible association between the Arg389Gly polymorphism and LVH among non-diabetic and diabetic acute myocardial infarction (AMI) survivors.

**Methods:**

The study population consisted of 452 AMI survivors, 20.6% of whom had diagnosed DM. Left ventricular parameters were measured with two-dimensional guided M-mode echocardiography 2-7 days after AMI, and the Arg389Gly polymorphism was determined using a polymerase chain reaction-restriction fragment length polymorphism assay.

**Results:**

The Arg389 homozygotes in the whole study population had a significantly increased left ventricular mass index (LVMI) when compared to the Gly389 carriers (either Gly389 homozygotes or Arg389/Gly389 heterozygotes) [62.7 vs. 58.4, respectively (p = 0.023)]. In particular, the Arg389 homozygotes displayed thicker diastolic interventricular septal (IVSd) measures when compared to the Gly389 carriers [13.2 vs. 12.3 mm, respectively (p = 0.004)]. When the euglycemic and diabetic patients were analyzed separately, the latter had significantly increased LVMI and diastolic left ventricular posterior wall (LVPWd) values compared to the euglycemic patients [LVMI = 69.1 vs. 58.8 (p = 0.001) and LVPWd = 14.2 vs. 12.3 mm (p < 0.001), respectively]. Furthermore, among the euglycemic patients, the Arg389 homozygotes displayed increased LVMI and IVSd values compared to the Gly389 carriers [LVMI = 60.6 vs. 56.3, respectively (p = 0.028) and IVSd = 13.1 vs. 12.0 mm, respectively (p = 0.001)]. There was no difference in the LVMI and IVSd values between the diabetic Arg389 homozygotes and Gly389 carriers.

**Conclusions:**

The data suggest an association between the β_1_AR Arg389Gly polymorphism and LVH, particularly the septal hypertrophy. The Arg389 variant appears to confer a higher risk of developing LVH than the corresponding Gly389 variant among patients who have suffered AMI. This association cannot be considered to be universal, however, since it does not appear to exist among diabetic AMI survivors.

## Background

Cardiac left ventricular hypertrophy (LVH) is an important risk factor for an adverse outcome in patients both with or without coronary artery disease [1]. Furthermore, LVH represents an independent risk factor for sudden cardiac death, congestive heart failure, coronary heart disease and stroke, and it has been associated with diabetes mellitus (DM) and glucose intolerance in several epidemiological investigations [2-6]. On the other hand, LVH can also be explained by genetic factors [7-9] and its heritability has been estimated to be 0.17-0.69 [10-12].

The beta-1 adrenergic receptor (β_1_AR) has been considered to represent a potential candidate gene for LVH [12-15]. This G protein-coupled receptor is the predominant beta adrenergic receptor in the heart (~ 70% β_1_AR and 30% β_2_AR), maintaining cardiac contractility in response to endogenous catecholamines [16]. The β_1_AR gene is localized to 10q24-26 [17] and was cloned in 1987 [18]. It encodes a 477-amino acid membrane protein that carries two common nonsynonymous single nucleotide polymorphisms, one in the extracellular amino terminus (Ser49Gly) and another in the proximal carboxyl terminus (Arg389Gly) [19]. The Arg389Gly polymorphic site lies within the putative G_s _coupling domain of the receptor [20]. It has thus been considered to have functional significance, because the positively charged arginine residue differs markedly from neutral glycine. Accordingly, the polymorphism has been shown to have an effect on signalling properties of the receptor [21].

The aim of our study was to investigate the possible association of the β_1_AR Arg389Gly polymorphism with various variables, including cardiac left ventricular parameters, among acute myocardial infarction (AMI) survivors in Northern Finland. We analyzed 452 patients, 20.6% of whom had diagnosed DM.

## Methods

### Patient population

The study population was recruited in 1996-2000. This single-center, prospective study, the Multiple Risk Factor Analysis Trial, was carried out at the Institute of Clinical Medicine, Department of Internal Medicine, Division of Cardiology, University of Oulu [22]. The aim of the study was to determine the prognostic power of several non-invasive risk markers of mortality among AMI survivors. A total of 452 consecutive series of patients who had undergone AMI were investigated for the β_1_AR Arg389Gly polymorphism. The patients were recruited to participate in the study during the first week after the AMI diagnosis, which was confirmed by using the contemporary guidelines at the beginning of the study. The exclusion criteria and qualifying diagnosis have been described previously [22].

### Biometric and laboratory methods

Height and weight were measured for each patient by a registered nurse. Body mass index (BMI) was obtained from the ratio of weight (kg) to height squared (m^2^). Blood pressure was obtained in a supine position by a registered nurse following a five minute resting period. Blood samples were collected from the patients after an overnight fast. The fasting glucose, total cholesterol, HDL cholesterol, LDL cholesterol and triglyceride levels were measured at the Institute of Diagnostics, Department of Clinical Chemistry at Oulu University Hospital using standard enzymatic methods.

### Genotyping

The β_1_AR Arg389Gly polymorphism was determined by using a polymerase chain reaction-restriction fragment length polymorphism assay, a modified PCR-technique described previously by Maqbool [19]. The restriction endonuclease Bcg I used in the protocol was from New England BioLabs (Beverly, MA, USA). The genotyping was performed in 2009 using DNA samples which had been stored at -20°C.

### Echocardiography and variable calculation

Left ventricular parameters were measured within 2-7 days after AMI. Echocardiographic studies were performed at the Institute of Clinical Medicine, Department of Internal Medicine, Division of Cardiology at Oulu University Hospital. Left ventricular internal dimension (LVID), interventricular septal thickness (IVS) and left ventricular posterior wall thickness (LVPW) were measured at end-diastole (d) and end-systole (s). The parameters were measured with two-dimensional guided M-mode echocardiography following the American Society of Cardiology/American Heart Association guidelines. Considering each parameter, three distinct measurements were done and the averages were calculated.

Left ventricular mass (LVM) was calculated from LVIDd, LVPWd and IVSd using the equation originally described by Devereux and colleagues [23]. This method gives values, which have been confirmed to be closely related to the necropsy left ventricular weight. To obtain LVMI, LVM was divided by height^2.7^.

### Data analysis

Statistical analyses were performed with SPSS 17.0/PASW 18.0 software packages and where appropriate, power calculations were performed with SamplePower 2.0. In the power calculations, alpha was set as 0.05. The Hardy-Weinberg equilibrium was tested with the Χ^2 ^test. Student's two-tailed t-test was used to compare the quantitative parameters (expressed as means ± S.D) between the groups, respectively. Categorical variables were compared using the Χ^2 ^analysis. Analysis of covariance (ANCOVA) was used for comparison of the echocardiographic variables between the genotype groups (expressed as means ± S.D). Using ANCOVA, the β_1_AR Arg389Gly polymorphism was set as a fixed factor. Age and BMI were added as co-variables to adjust the results. P < 0.05 was considered statistically significant. Nominal p values are presented throughout, i.e. without correction for multiple testing, as recommended by Rothman [24].

## Results

### Characteristics of the AMI population

The main clinical and biochemical data of the study population are shown in Table 1, in which the euglycemic and diabetic patients have been analyzed separately. As expected, the euglycemic and diabetic subgroups differed in many clinical aspects in addition to the history of DM and fasting glucose. There were significantly more women among the diabetic group, and the mean age as well as BMI and fasting triglycerides were higher as compared to the euglycemic group. Among the diabetic patients, the prevalence of congestive heart failure was significantly greater, and therefore the use of diuretics and digoxin, and partly angiotensin-converting enzyme inhibitors or sartans, was more frequent than among the euglycemic individuals. Table 2 shows the echocardiographic parameters, again analyzed separately for the euglycemic and diabetic subgroups. As expected [2-6], LVMI, and also LVPWd values were significantly higher among the diabetic patients when compared to the euglycemic individuals. Power calculations revealed the power to detect differences between the euglycemic and diabetic patients 100% for both LVMI and LVPWd.

**Table 1:** Clinical and biochemical characteristics of the euglycemic and diabetic patients in the study population.

	Total n = 452	Euglycemic patients n = 359 (79.4%)	Diabetic patients n = 93 (20.6%)	p value
Gender (male/female)	354/98 (78.3/21.7%)	289/70 (80.5/19.5%)	65/28 (69.9/30.1%)	0.027
Age (years)	60.8 ± 9.7	60.0 ± 9.5	63.8 ± 9.8	0.001
BMI	27.5 ± 3.8	27.3 ± 3.6	28.4 ± 4.4	0.014
Height (cm)	170.4 ± 8.4	170.6 ± 8.1	169.4 ± 9.4	NS
Weight (kg)	79.9 ± 13.2	79.6 ± 13.2	81.2 ± 13.2	NS
SBP (supine mmHg)	123.2 ± 19.1	122.7 ± 18.7	125.7 ± 20.7	NS
DBP (supine mmHg)	80.2 ± 10.9	80.5 ± 10.9	79.0 ± 11.2	NS
Congestive heart failure	45 (10.0%)	16 (4.5%)	29 (31.2%)	< 0.001
Concomitant medications				
Acetylsalicylic acid	392 (86.7%)	317 (88.3%)	75 (80.6%)	NS
Beta blocker	440 (97.3%)	348 (96.9%)	92 (98.9%)	NS
ACE inhibitor/sartan	179 (37.6%)	123 (34.3%)	47 (50.5%)	0.004
Diuretic	87 (19.2%)	50 (13.9%)	37 (39.8%)	< 0.001
Calcium channel blocker	26 (5.8%)	21 (5.8%)	5 (5.4%)	NS
Statin	176 (38.9%)	140 (39.0%)	36 (38.7%)	NS
Warfarin	36 (8.0%)	28 (7.8%)	8 (8.6%)	NS
Digoxin	19 (4.2%)	10 (2.8%)	9 (9.7%)	0.003
Amiodarone	6 (1.3%)	6 (1.7%)	0 (0%)	NS
Fasting glucose (mmol/l)	6.4 ± 2.1	5.9 ± 1.4	8.3 ± 2.9	< 0.001
Total cholesterol (mmol/l)	5.4 ± 3.4	5.4 ± 3.7	5.3 ± 1.2	NS
HDL cholesterol (mmol/l)	1.1 ± 0.3	1.2 ± 0.3	1.1 ± 0.3	NS
LDL cholesterol (mmol/l)	3.4 ± 0.9	3.4 ± 0.9	3.4 ± 1.1	NS
Triglycerides (mmol/l)	1.6 ± 0.9	1.5 ± 0.9	2.0 ± 1.1	< 0.001

Student's two-tailed t-test (quantitative variables) or Χ^2 ^analysis (categorical variables) was performed to compare the populations. BMI, body mass index; SBP, systolic blood pressure; DBP, diastolic blood pressure; ACE, angiotensin-converting enzyme; NS, not significant. Data for continuous variables are presented as mean ± S.D. Categorical variables are presented as number of subjects and as percentages (in parentheses).

**Table 2:** Echocardiographic variables of the euglycemic and diabetic patients in the study population.

	Total n = 452	Euglycemic patients n = 359 (79.4%)	Diabetic patients n = 93 (20.6%)	p value
LVMI	60.9 ± 19.4	58.8 ± 18.3	69.1 ± 21.7	0.001
IVSd (mm)	12.8 ± 3.0	12.7 ± 2.8	13.4 ± 3.3	NS
LVIDd (mm)	49.8 ± 7.1	49.6 ± 7.1	50.5 ± 7.1	NS
LVPWd (mm)	12.7 ± 3.6	12.3 ± 2.9	14.2 ± 5.3	< 0.001
EF	46.0 ± 8.7	46.3 ± 8.4	44.8 ± 9.4	NS

ANCOVA was performed to compare the groups. The polymorphism was set as a fixed factor. Age and BMI were added as co-variables to adjust the results. LVMI, left ventricular mass index; IVSd, interventricular septal thickness at end-diastole; LVIDd, left ventricular internal dimension at end-diastole; LVPWd, left ventricular posterior wall thickness at end-diastole; EF, ejection fraction. Data are presented as mean ± S.D.

### Arg389Gly allele and genotype frequencies

The β_1_AR genotype at codon 389 was determined in 452 AMI survivors. The allele frequencies were 0.75 for Arg389 and 0.25 for Gly389. These values were in accordance with frequencies observed in other Caucasian populations [25-27]. The frequencies of subjects homozygous for the Arg389 or Gly389 alleles were 0.57 and 0.07. The genotype frequencies were also in line with previous European studies [26,27]. The study population was in Hardy-Weinberg equilibrium (Χ^2 ^= 0.817).

### Arg389Gly polymorphism and echocardiographic parameters

The echocardiographic parameters according to the Arg389Gly polymorphism in the whole study population are listed in Table 3. The Arg389 homozygotes were found to have significantly increased mean LVMI when compared to the Gly389 carriers (p = 0.023). In particular, the Arg389 homozygotes displayed thicker IVSd measures when compared to the Gly389 carriers (p = 0.004). Power calculations indicated that the study sample had 67% power to detect differences in LVMI between the Arg389 homozygotes and the Gly389 carriers. Furthermore, power calculations showed that the required sample size for the achievement of power equal to 80% would be 580 subjects. For IVSd, the power was calculated to be 90%.

**Table 3:** Echocardiographic variables in the whole study population according to the β_1_AR Arg389 homozygotes and Gly389 carriers.

	Total n = 452		
	Arg homozygotes n = 258 (57.1%)	Gly carriers n = 194 (42.9%)	p value
LVMI	62.7 ± 19.1	58.4 ± 19.8	0.023
IVSd (mm)	13.2 ± 3.1	12.3 ± 2.7	0.004
LVIDd (mm)	49.8 ± 7.3	49.9 ± 6.7	NS
LVPWd (mm)	12.9 ± 3.6	12.5 ± 3.6	NS
EF	45.9 ± 8.7	46.2 ± 8.7	NS

ANCOVA was performed to compare the genotype groups. The polymorphism was set as a fixed factor. Age and BMI were added as co-variables to adjust the results. For abbreviations, see Table 2. Data are presented as mean ± S.D.

Table 4 shows the echocardiographic variables according to the Arg389Gly polymorphism analyzed in the euglycemic patients. The Arg389 homozygotes displayed increased LVMI values compared to the Gly389 carriers (p = 0.028). Again, particularly the IVSd values were higher among the Arg389 homozygotes compared to the Gly389 carriers (p = 0.001). The calculated powers were 62% for LVMI (required sample size for the achievement of 80% power would be again 580 subjects) and as high as 100% for IVSd.

**Table 4:** Echocardiographic variables in the euglycemic patients according to the β_1_AR Arg389 homozygotes and Gly389 carriers.

	Euglycemic patients n = 359 (79.4%)		
	Arg homozygotes n = 203 (56.5%)	Gly carriers n = 156 (43.5%)	p value
LVMI	60.6 ± 17.5	56.3 ± 19.2	0.028
IVSd (mm)	13.1 ± 3.0	12.0 ± 2.4	0.001
LVIDd (mm)	49.4 ± 7.3	49.9 ± 6.9	NS
LVPWd (mm)	12.6 ± 3.0	12.0 ± 2.8	NS
EF	46.6 ± 8.5	46.0 ± 8.4	NS

The analyses were performed and data are presented as described in Table 3. For abbreviations, see Table 2.

Table 5 shows that there was no difference in LVMI and IVSd values between the diabetic Arg389 homozygotes and Gly389 carriers. Ejection fraction (EF) was slightly smaller among the Arg389 homozygotes when compared to the Gly389 carriers (p = 0.049), but this finding is most likely only random variation because of the small sample size.

**Table 5:** Echocardiographic variables in the diabetic patients according to the β_1_AR Arg389 homozygotes and Gly389 carriers.

	Diabetic patients n = 93 (20.6%)		
	Arg homozygotes n = 55 (59.1%)	Gly carriers n = 38 (40.9%)	p value
LVMI	70.3 ± 22.8	67.2 ± 20.3	NS
IVSd (mm)	13.4 ± 3.3	13.5 ± 3.3	NS
LVIDd (mm)	51.1 ± 7.5	49.6 ± 6.5	NS
LVPWd (mm)	13.9 ± 5.2	14.6 ± 5.5	NS
EF	43.3 ± 9.0	46.9 ± 9.8	0.049

The analyses were performed and data are presented as described in Table 3. For abbreviations, see Table 2.

## Discussion

The main finding of our study is the apparent association of the β_1_AR Arg389 homozygotes with LVH among euglycemic AMI survivors, and that this association does not appear to exist among diabetic AMI survivors. Analyzing 452 Finnish patients who had undergone AMI, 79.4% of whom were euglycemic and 20.6% diabetic, we were able to show that the β_1_AR Arg389 homozygotes had significantly higher LVMI when compared to the Gly389 carriers (either Gly389 homozygotes or Arg/Gly389 heterozygotes). The same applied also to IVSd, and this association was even stronger. Our result is logical based on previous reports on the functional properties of the two receptor variants. The β_1_AR Arg389 variant has been shown to display markedly increased coupling to G_s _and stimulation of adenylyl cyclase in response to the agonist isoproterenol *in vitro *as compared to the Gly389 receptor form [21]. Gly389 is thought to disrupt the predicted alpha-helical region of the cytoplasmic carboxyl terminus of the receptor, which may cause a less favorable receptor conformation for coupling to G_s _[28]. Thus, the β_1_AR Arg389 variant can be considered as a "gain-of-function" receptor form.

It is well known that prolonged activation of the β_1_AR leads to worsening of cardiac function. As a consequence, β_1_AR expression is down-regulated and its coupling to adenylyl cyclase is desensitized [29]. The Arg389Gly polymorphism appears to modulate this pathological situation: Mialet Perez *et al*., who used transgenic mice, showed that the Arg389 variant is impaired in down-regulation, which most likely represents a protective event in the failing myocardium. Furthermore, the young Arg389 mice were found to have enhanced receptor function and heart contractility compared to the Gly389 carriers, whereas the older Arg389 mice displayed a phenotypic switch with decreased signalling to adenylyl cyclase and contractility compared to the corresponding Gly389 mice [30]. In line with these *in vivo *studies, Rathz *et al*. have shown that the β_1_AR Arg389 variant undergoes less agonist-promoted desensitization *in vitro *compared to its allelic counterpart [31]. Recently, Lewin and colleagues have thrown light on the mechanism underlying the β_1_AR-induced cardiac damage by showing that inactivation of the cyclic AMP response element modulator (CREM) rescued the β_1_AR-overexpressing mice from cardiomyocyte hypertrophy, fibrosis, and left ventricular dysfunction [32]. It would be interesting to know, whether there is a difference in CREM expression and/or function between individuals carrying either the Arg389 or Gly389 variant - a highly speculative possibility worth investigating.

Our results support the notion that there is a direct genetic impact of the β_1_AR Arg389 homozygosity on the left ventricular structure. We cannot, however, rule out the possibility that the observed increases in LVMI and IVSd among the β_1_AR Arg389 homozygotes were related to hypertension because LVH partly represents an adaptive response to hypertension [33]. In previous studies it has been shown that the β_1_AR Arg389 homozygotes have an increased risk to develop essential hypertension among Scandinavians [34] and Chinese [35]. We, nevertheless, did not detect higher blood pressure values among the Arg389 homozygotes as compared to the Gly389 carriers in our study population (data not shown). This negative finding most likely results from the anti-hypertensive medication of the patients that took place after AMI and hospitalization and/or from the relatively small sample size.

To our knowledge, there are four previous studies concerning the β_1_AR Arg389Gly polymorphism and left ventricular structure. In accordance with our study, Fu *et al*. described a significant association between the β_1_AR Arg389 variant and LVH in Chinese hypertensive individuals in two independent populations (n = 2417 and n = 327) [13]. In contrast, no relationship between LVM and the β_1_AR Arg389Gly polymorphism was found in a study population consisting of 110 healthy Caucasian twin pairs (n = 220) [12]. In a third study by Stanton and colleagues, the Gly389 homozygotes were observed to have higher LVM when compared to the Arg389 homozygotes among 249 Caucasian patients suffering from renal failure [14]. This study consisted of a very specific population: each individual had a proven renal disease, 37% of whom were on renal replacement therapy. This may explain the result that seems physiologically counter-intuitive. In the fourth study, Meyers *et al*. who studied African American siblings suffering from hypertension found no significant association between the β_1_AR Arg389Gly polymorphism and LVMI but reported that the Gly389 allele was significantly associated with a higher mean relative wall thickness compared to the Arg389 form [15]. This association was not replicated in another study group consisting of a Hispanic cohort. It is important to note that the Gly389 allele is more frequent in black populations [15,25], which may explain the divergent findings.

When we analyzed the euglycemic and diabetic patients of our study group separately, we did not find any association between the β_1_AR Arg389Gly polymorphism and LVH among the diabetic patients. The negative result can be considered to arise from an independent association between DM and LVH [2-6], which is presumably stronger than the association of the β_1_AR Arg389Gly polymorphism with LVH. Therefore the assumed stronger effect of DM can be expected to mask the weaker effect of the polymorphism on the left ventricular structure. Interestingly, the association between DM and LVH seems to be stronger in women than in men [2,5,6]. In our study population the proportion of women was significantly higher among the diabetic subgroup as compared to the non-diabetics one (30.1/19.5%), supporting the hypothesis of the aforementioned "masking effect" of DM. Furthermore, the β_1_AR expression has been shown to be markedly decreased in the hearts of diabetic patients (atrial appendages) [36], which may also have had an impact on our results.

## Limitations

Evaluating left ventricular parameters from post-AMI patients with two-dimensional guided M-mode echocardiography is not always reliable if the infarction happens to be in the area where the measures are being taken from. Determining the EF with the M-mode device has also limitations, because the systolic function is measured only from a single point of the left ventricle. Furthermore, in our study, echocardiography was performed within one week after AMI, and consequently hyperaemia may have contributed to the measurements in addition to the left ventricular dysfunction and remodeling. Additionally, the amount of diabetic patients was rather small in our study sample, which causes certain limitations to the statistic analyses.

## Conclusions

As a conclusion, we show here that LVMI and IVSd values are significantly higher among AMI survivors who are homozygous for the β_1_AR Arg389 variant compared to the AMI patients carrying either one or two copies of the Gly389 allelic form of the β_1_AR. The β_1_AR Arg389 variant thus seems to confer higher risk of developing LVH. In addition, we show that the aforementioned association is not universal, since it does not exist among diabetic AMI survivors. We hypothesize that this negative finding is caused by the strong association between DM and LVH, which may mask the presumably weaker effect of the β_1_AR Arg389 variant on the left ventricular structure.

## Abbreviations

ANCOVA: analysis of covariance; AMI: acute myocardial infarction; β_1_AR: beta-1 adrenergic receptor; BMI; body mass index; DM: diabetes mellitus; EF: ejection fraction; IVSd; interventricular septal thickness at end-diastole; LVH: left ventricular hypertrophy; LVIDd: left ventricular internal dimension at end-diastole; LVM: left ventricular mass; LVMI: left ventricular mass index; LVPWd: left ventricular posterior wall thickness at end-diastole

## Competing interests

The authors declare that they have no competing interests.

## Authors' contributions

AEH carried out the genotyping of patients, performed the statistical analyses and drafted the manuscript. JMT performed the echocardiography. JMJ, KSK and HVH critically reviewed the manuscript. UEP-R critically reviewed the manuscript and helped to draft it. All authors read and approved the final manuscript.
